# A Case of Tubercular Meningitis

**Published:** 1893-10

**Authors:** Allen J. Smith

**Affiliations:** Professor of Pathology and Lecturer on Mental and Nervous Diseases in the Medical Department of the University of Texas, Galveston


### TEXAS MEDICAL JOURNAL.

ESTABLISHED JULY, 1885.

PUBLISHED MONTHLY.—SUBSCRIPTION $2.00 A YEAR.

Vol. IX. AUSTIN, OCTOBER, 1893. No. 4.

### Original Contributions.

For Texas Medical Journal.

### A CASE OF TUBERCULAR MENINGITIS.

BY ALLEN J. SMITH, M. D., GALVESTON,
Professor of Pathology and Lecturer on Mental and Nervous Diseases in the Medical Department of the University of Texas, Galveston.

ALTHOUGH as an affection of early life tubercular meningitis is not uncommon, it is a comparatively infrequent form of tuberculosis in the adult; and this circumstance has suggested the propriety of recording the following case. In children tubercular meningeal invasion is, after glandular tuberculosis, one of the most common varieties of the disease; but in the adult it becomes much less frequent in proportion to tuberculosis of other portions of the body. It by no means follows in the adult, just as it does not follow in the child, that there should exist an advanced grade of tuberculosis elsewhere to cause secondary meningeal invasion. On the contrary, the clinical rule seems to be that tubercular meningitis occurs in individuals with comparatively slight tubercular processes in other parts of the body. The path of transmission of the tubercular virus to the contents of the skull cavity is the arterial stream, the currents of venous blood and lymph having the opposite direction of flow and convection. The involvement of the membranes or of the brain substance is therefore often part of a general tubercular disease; and many of the symptoms of acute general miliary tuberculosis are to be regarded as dependent upon the involvement of these parts of the general nervous system. The localized partial palsies often met in tubercular individuals, involving some group of muscles here or there on one side of the body, perhaps in the limb or in the arm, are sometimes at least due to aneurismal formation in the small arteries of the brain substance or to tubercular masses along the walls of these vessels; but may also depend upon localized meningitis with cortical invasion. Such processes are part of a much more chronic course of the affection, however, as a rule, than is to be expected in acute general tuberculosis; and are moreover more slow than the ordinary form of tubercular meningitis, so frequently fatal, into which these localized involvements may merge. There is no definite circumstance which may be recognized as determining in what case of tuberculosis there will or will not develop a secondary tubercular meningitis; but it is not improbable that a hereditary tendency exists, inasmuch as the condition has frequently been encountered in several instances in the same family. This fact is illustrated in the following instance, which in many points is an excellent example of the symptomatology of the affection.

J. A., a white male, aged about twenty-seven years, unmarried, a student of medicine, a native of Texas, came to the writer for a diagnosis of his condition in January of the present year. He was one of four children, two of whom were dead, one from violence, the other from tubercular meningitis. His father had died from an obscure cardiac affection in old age, and his mother from an unknown cause during his childhood. He had passed through childhood without serious illness, had had several attacks of malarial fever during his youth; and about five or six years ago had had a rectal fistula, which, after operation, healed readily. About the same time he had a persistent cough and had lost flesh, but these symptoms disappeared without special treatment other than a prolonged sojourn in the country after the relief from the rectal fistula. When the patient came under observation he stated that for several months he had experienced from time to time a pleuritic-likepain in the right hypochondrium and axillary region, had a dry, irritative cough, and for some weeks had been losing flesh and color. He had been very irregular in his diet for some months and his appetite was capricious. No organic disease was detected other than a decided thickening of the pleura in the lower portion of the right side of the chest, most marked in the axillary line and extending up to about the seventh or eighth rib in this region. There was a certain amount of pulmonary infiltration, and some catarrhal involvement of the bronchial tubes. The entire right lung at this time, and the upper portion of the left lung as well, had an altered respiratory rhythm, expiration being prolonged and inspiration occasionally "catchy." No bacilli were detected in the sputum on careful examination. The patient had been taking capsules of creasote and morrhuol by the advice of another physician he had consulted, and he was urged to pay attention to his dietary, proper suggestions being made in that direction. Cod liver oil and whisky were advised, with counter-irritation over the pleuritic area; and a stomachic tonic was prescribed. Little or no change, save the appearance of a similar pleuritic patch over the lower portion of the left lung anteriorly, and the natural increase in extent and definiteness of the signs in the right side, was to be noted in the next three months. During this time he had made several visits to his home in the interior of the State, and at first for a time was apparently benefited. He became imbued, however, with the idea that a hectic fever, which appeared about the end of February, was due to some hepatic disorder, and while in the country began a systematic and persistent dosage of himself with calomel, salts, podophyllin and similar remedies. He was examined several times by the writer after January, and each time was advised to carry out energetically anti-tubercular measures, and was assured of the integrity of the liver. He persisted, however, in his belief in the existence of an hepatic affection and doubted the serious disease of the lungs, meantime continuing to physic himself and irritate his alimentary canal by violent purgatives. The man was of a highly neurotic family, and was naturally of a hypochondriacal nature, a tendency which easily became intensified by his medical studies and which during his illness was a conspicuous element.

In May, about four weeks after the last examination, Mr. A. followed the writer to Southwestern Texas, near San Antonio, a district well known for the dryness and purity of its atmosphere. Examination of the chest at this time revealed decided involvement of both lungs; and the cough, now violent and frequent in the morning and evening, was beginning to be fruitful. The patient was at once placed upon the fullest dietary, cod liver oil, iron, quinine and strychnine administered, the chest was freely painted with iodine, and for a few days there was an apparent improvement. The patient was highly hysterical by this time, from the intensity of his hypochondriasis; was fearful of death, yet refused to accept the existence of tuberculosis, and secretly administered calomel and podophyllin "to act on his liver." A week after coming into the Southwestern district he spat up several mouthfuls of blood after coughing, and from the moment of the appearance of the first blood apparently lost all hope of his recovery. He at once went to bed, where he lay, in a more or less apathetic condition, for more than a week, only aroused to interest by conversation upon his own condition, or upon some topic connected with certain stirring episodes in his past life. Now for the first time appeared the signs of the meningitis, at first unappreciated and confused with the hypochondriacal symptoms which had been present for the greater part of his illness. He began to complain of headache of general distribution, unaffected by medication, and rapidly growing worse, so as to keep him awake much of the night. The fever, which had previously been usually perceptible only in the afternoon and evening, became more or less constant, ranging from 100° F. to 102.5° F. Vomiting became a prominent symptom, occurring at almost any time, but almost uniformly after the patient had eaten. These symptoms were interpreted by the patient as evidence of the correctness of his view of implication of the liver, and he redoubled his efforts at purgation, only succeeding in rendering the stomach and intestines irritated, and in reducing his already diminished flesh and strength. The headache increased in severity, and became localized in its intensity in the back part of the head.

The patient, in the third week after coming under constant care, and about eight days after the first appearance of meningeal symptoms, began to exhibit a tendency to retraction of the head, and manifested an ataxic aphasia. This aphasia was not constant, however, at first, and could be suspended by will of the patient, or would apparently spontaneously disappear when the patient's interest could be aroused to a conversation. The cardiac rate at this peroid diminished in rapidity, the beats dropping from an increased rate of 80 per minute to less than 60, and sometimes less than 50, per minute. The senses of sight and hearing were but little affected, but to a slight degree the eyes became impatient of bright light, and the ears of shrill sounds. A tremor quite like that met with in delirium tremens, became manifest, and persisted to the end of the case.

These symptoms, headache, vomiting, retraction of the head, aphasia, cardiac inhibition, irritability of the organs of sight and hearing, with the restlessness, insomnia, disturbed, dreamful sleep, and tremor, mark the first period in the course of the disease, that of cerebral irritation. During this period, there occurred at several times, symptoms of collapse, relieved after vomiting, during which the fear of impending death was most distressing and alarming to the patient. The bowels were confined, except wnen moved by purgative medicines. The symptoms of this period, quite clear in the light of the subsequent course, were not appreciated at the time, being confused with the many and very pronounced signs of hysteria. This period of irritation may be said to have lasted in its intensity about eight days, or about sixteen or eighteen from the time when recognizable symptoms first manifested themselves. Its inception was by no means clearly defined, the malaise, the tired, unrested feeling, the tendency to prostration, of the primary affection gradually merging into the stage which exhibited a definite symptomatology. Toward the close of this period the symptoms were very pronounced, and the diagnosis of meningeal invasion was seriously entertained, although not positively, because of the fear of confusion with the hysteria, which had so decidedly shown after the hemorrhage. The ability of the patient to overcome his ataxic speech, the patient's exaggeration of his inability to sleep and to eat, his detected habit of secretly administering purgatives to himself, his apparent magnification of comparatively slight symptoms, all tended to obstruct the clearer judgment of the case; and it was only when this period of irritation was at its greatest intensity and was being succeeded by the stage of depression, the so-called "paralytic" stage, that the patient's condition was fully realized. The restlessness and sleeplessness were succeeded by drowsiness. The patient slept the greater part of the time, but his sleep was ever harassed by dreams. One persistent dream complained of was that the patient was the owner of a herd of black ponies, which he was training for the purpose of placing them upon the market. Sometimes the idea about these ponies occurred to him in his waking moments, as delusions. His headache, so intense in the last days of the period of irritation that the patient tossed about on his bed and rolled his head from side to side, often drawing his face into an apparently involuntary grimace, gradually subsided, only a dull, general pain in the head remaining. The retraction of the head became more marked, and the nuchal muscles became sore and painful. The drowsiness became more and more decided, and even when awake he seemed to be in a daze. His mentality gradually showed impairment. He frequently would converse with some absent acquaintance, and suddenly arouse to the knowledge that there was no one present. He had hallucinations of hearing, and was the subject of mild depressive, persecutory delusions. On several occasions he seemed to have suicidal tendencies, and a watch was systematically kept over his actions. His movements became slow, and often purposeless. Gradually, in his worse moments, in addition to the aphasia, his language became disconnected, and his ideas incoherent.

Yet, at any time, if tnoroughly aroused from his dazed, stupid state, he was able to intelligently and correctly answer inquiries. Often his sentences, badly articulated, would be left unfinished, and in the midst of an answer to some query, he would stop and stare vacantly into space. The same incoherence and lack of sequence could be noted in a few letters he attempted to write, the tremulousness of his hand rendering the chirography, moreover, almost illegible. At times he would talk to himself for ten or fifteen minutes, or longer, without any apparent appreciation of his surroundings. His habits of neatness became neglected; he would dress himself, leaving the clothes unbuttoned, and the shoes unlatched. At the table, while his appetite improved, he became careless and untidy. He was exceedingly forgetful; he would sit looking blankly at a letter, which he might have read in a few moments when in health, for half an hour or more, and in the end would fail to give a connected account of its contents. His volition, as well as the other mental faculties, suffered, and in the end he depended almost entirely upon the will of others to guide his actions. Along with these changes there progressed rapidly a loss of motor power; and after three or four days, from the commencement of the depressive stage, he was unable to move about his room without aid. He rapidly lost flesh as well. There were no appreciable focal symptoms other than the alteration of speech; and at no time, while under observation, were there any distinct palsies. In this condition the patient was sent to his home to die, the termination coming on four days later, or about eight or nine days from the beginning of the depressive stage, and about three weeks, or slightly more, from the time of the first symptom-manifestation of the meningeal trouble. The symptoms immediately preceding his death were, as related to the writer, but an intensification of those narrated above. The stupor became more and more pronounced, and the last two days of his life the patient was comatose. The cephalalgia persisted, and even in his unconscious moments the patient would pass his hand tremulously to his head, as if in pain. Death occurred finally from cardiac failure.

During the course of the meningeal affection, there was a notable abatement of the pulmonary symptoms. The cough and expectoration became very much diminished, and the physical signs of the catarrhal involvement of the bronchial mucous membrane were decidedly improved. To a certain extent, it is likely that the appreciation of the bronchial irritation was dulled by the nervous condition; but the fuller and slower respirations, which were present after the meningitis became pronounced, and the absence of cough, could scarcely have been due to this fact entirely, especially when one recalls the improvement in the physical signs. During the depressive stage the nausea and vomiting, which had been so severe at first, were much relieved; and in spite of his condition, until he left Southwest Texas, the patient ate with apparent relish. His bowels, during this same period, were confined, the patient having but one or two movements. The slowness of the cardiac beat, which was so well marked in the former stage, was not present with uniformity after the period of depression had set in, intervals of increase of frequency appearing irregularly. As long as he was under the writer's observation, there was no inability to attend to his private functions. The urine was free and uniformly of a high color and strong odor. The symptoms of the second stage may thus be briefly stated in resumé in contrast with those of the period of the meningeal irritation: headache, of a less severe and duller character, but quite as persistent as in the first stage; drowsiness, stupor, and eventually coma, replacing the restlessness of the first stage; failure of all the mental powers, gradually, but to an advanced degree; cessation of vomiting, and the appearance of tachycardia; rapid and marked loss of strength and flesh.

It is usual to add a third stage to the two described, that of intracranial pressure, placed between the stages of irritation and depression. In this case the last named stage would, however, embrace only the last few hours of life, those of profound coma, and it has seemed best, therefore, to separate the course into only two stages, irritative and despressive. It is difficult to realize why a division of what is pathologically but a single stage should be made, the later phenomena of the course being but the natural development from an intensification of the intracranial pressure and degenerative changes which are responsible for all the symptoms following those of the irritative period.

No autopsy was performed in the above instance.

